# Supplementary material for: Antibacterial compounds against non-growing and intracellular bacteria
Source: NPJ Antimicrob Resist. 2025 Apr 11;3:25. doi: 10.1038/s44259-025-00097-0 (PMC11992225; doi:10.1038/s44259-025-00097-0)
Supplement: Supplementary file 4 — Supplementary information [file 44259_2025_97_MOESM4_ESM.pdf]

Supplementary Information

Antibacterial Compounds Against Non-Growing and Intracellular Bacteria

Niilo Kaldalu[a]*, Normunds Bērziņš[a], Stina Berglund Fick[b], Atin Sharma[c,d], Naomi Charlotta Andersson[a], Jüri Aedla[a], Mariliis Hinnu[a], Andrea Puhar[c,d,e], Vasili Hauryliuk[a,f,g,h*], Tanel Tenson[a]*

[a]Institute of Technology, University of Tartu, Tartu, Estonia

[b]Chemical Biology Consortium Sweden, Umeå University, Umeå, Sweden

[c]Department of Molecular Biology, Umeå University, Umeå, Sweden

[d]The Laboratory for Molecular Infection Medicine Sweden (MIMS), Umeå, Sweden

[e]Wellcome-Wolfson Institute for Experimental Medicine (WWIEM), School of Medicine, Dentistry and Biomedical Sciences, Queen's University, Belfast, United Kingdom

[f]Department of Experimental Medical Science, Lund University, Lund, Sweden

[g]Science for Life Laboratory, Lund, Sweden

[h]Virus Centre, Lund University, Lund, Sweden

*Address correspondence to Niilo Kaldalu, niilo.kaldalu@ut.ee, Vasili Hauryliuk, vasili.hauryliuk@med.lu.se or Tanel Tenson, tanel.tenson@ut.ee

Supplementary Table 1. Chemical and physical properties of the hit compounds.

Individual, median and quartile values of the characteristics of the hit compounds versus median and quartile values of the Prestwick collection.

	COMPOUND	molecular weight (g/mol)	XLogP	TPSA (Å²)	Complexity	screen conc. (20 µM as µg/ml)
Anti-cancer	Bleomycin	1415.6	-7.5	685	2580	28.3
	Degrasyn	384.3	4.3	65.8	490	7.7
	Evofosfamide	449.04	0.6	114	374	9.0
	Echinomycin	1101.3	2.7	352	2200	22.0
	Mitomycin C	334.3	-0.4	147	757	6.7
	Plicamycin	1085.1	0.6	358	1940	21.7
	Satraplatin	502.3	NA	102	142	10.0
Disinfect ant	Alexidine	508.8	4.3	178	601	10.2
	Chlorhexidine	505.4	0.1	178	649	10.1
	Octenidine	550.9	11	31.2	691	11.0
Fluoroquinolone	Besifloxacin	393.8	1.1	86.9	656	7.9
	Gemifloxacin	389.4	-0.7	121	725	7.8
	Moxifloxacin	401.4	0.6	82.1	727	8.0
	Ofloxacin	361.4	-0.4	73.3	634	7.2
	Prulifloxacin	461.5	1	125	930	9.2
	Sitafloxacin	409.8	0.7	86.9	761	8.2
	Sparfloxacin	392.4	0.1	98.9	691	7.8
	Sarafloxacin	385.4	0.3	72.9	645	7.7
	Trovafloxacin	416.4	0.3	99.8	770	8.3
	Tosufloxacin	404.3	0.4	99.8	708	8.1
	Ulifloxacin	349.4	0.3	98.2	607	7.0
	Ciprofloxacin	331.3	-1.1	72.9	571	6.6
	Clinafloxacin	365.8	0.4	86.9	626	7.3
	Difloxacin	399.4	1.6	64.1	672	8.0
	Danofloxacin	357.4	-0.3	64.1	679	7.1
	Enrofloxacin	359.4	-0.2	64.1	613	7.2
	Finafloxacin	398.4	-0.7	106	806	8.0
	Gatifloxacin	375.4	-0.7	82.1	653	7.5
	Garenoxacin	426.4	2.1	78.9	771	8.5
Macrolide	Josamycin	828.0	2.9	206	1390	16.6
	Midecamycin	814	2.6	206	1360	16.3
	Rosamicin	581.7	2.3	135	941	11.6
	Solithromycin	845	4.3	198	1530	16.9
	Tylosin	916.1	1	239	1560	18.3
PML*	Valnemulin	564.8	5.3	144	969	11.3
Rifamycin	Rifampicin	822.9	4	217	1750	16.5
	Rifabutin	847.0	6.3	206	1870	16.9
	Rifapentine	877.0	5.8	217	1870	17.5
	FCE-22250	834.9	5.5	229	1660	16.7
	QUARTILES					
Hit compounds	Median	426.4	0.65	106	727	
	25% percentile	384.3	0.025	78.9	634	
	75% percentile	822.9	3.175	206	1390	
Prestwick collection	Median	311.4	2.4	68.35	405	
	25% percentile	249.1	0.7	43.1	276.3	
	75% percentile	392.5	3.7	104	602.5	

* pleuromutilin
NA – not applicable

Supplementary Table 2. CPEC primers

Primer	Sequence	Template
Vector		
term_vector_fwd	CAATATGGTGAGCAAGGGCGAGG	pSC101-GFP-mScarlet-I[1]<br><br>pAED1 (this study)
promoter_vector_rev	CTGTCAGGTCATTTCCAAGCTTGTCGA	
TestprimerAmp_rev	GACACGGAAATGTTGAATAC	
AmpCPEC_fwd	GGAAGAGTATGAGTATTCAACATTTCCGTGTC	
trpT terminator insert		
term_cpec_insert_fwd	CAACCGCAGTGAGTGAGTCTG	pSC101-CAM-bioreporter[2]
term_cpec_insert_rev	CCTCGCCCTTGCTCACCATATTGTGGTCAGTCATTTCCAAGCTTGTCGACCTGC	
cda promoter insert		
pcda_insert_fwd	TCGACAAGCTTGGAAATGACCTGACAGCGCTCTTCGGCTTCGGTCA	pANO1::*cda'*[3]
pcda_insert_rev	CCTCGCCCTTGCTCACCATATTGCACCTCCTTGACTTTTAAAACAATGCGTTAAAAACAACAAAC	

1. Hinnu, M., Putrinš, M., Kogermann, K., Kaldalu, N. & Tenson, T. Fluorescent reporters give new insights into antibiotics-induced nonsense and frameshift mistranslation. *Sci. Rep.* **14**, (2024).

2. Preem, L. *et al.* Monitoring of Antimicrobial Drug Chloramphenicol Release from Electrospun Nano- and Microfiber Mats Using UV Imaging and Bacterial Bioreporters. *Pharm. 2019, Vol. 11, Page 487* **11**, 487 (2019).

3. Norman, A., Hansen, L. H. & Sørensen, S. J. Construction of a ColD cda promoter-based SOS-green fluorescent protein whole-cell biosensor with higher sensitivity toward genotoxic compounds than constructs based on recA, umuDC, or sulA promoters. *Appl. Environ. Microbiol.* **71**, 2338–2346 (2005).

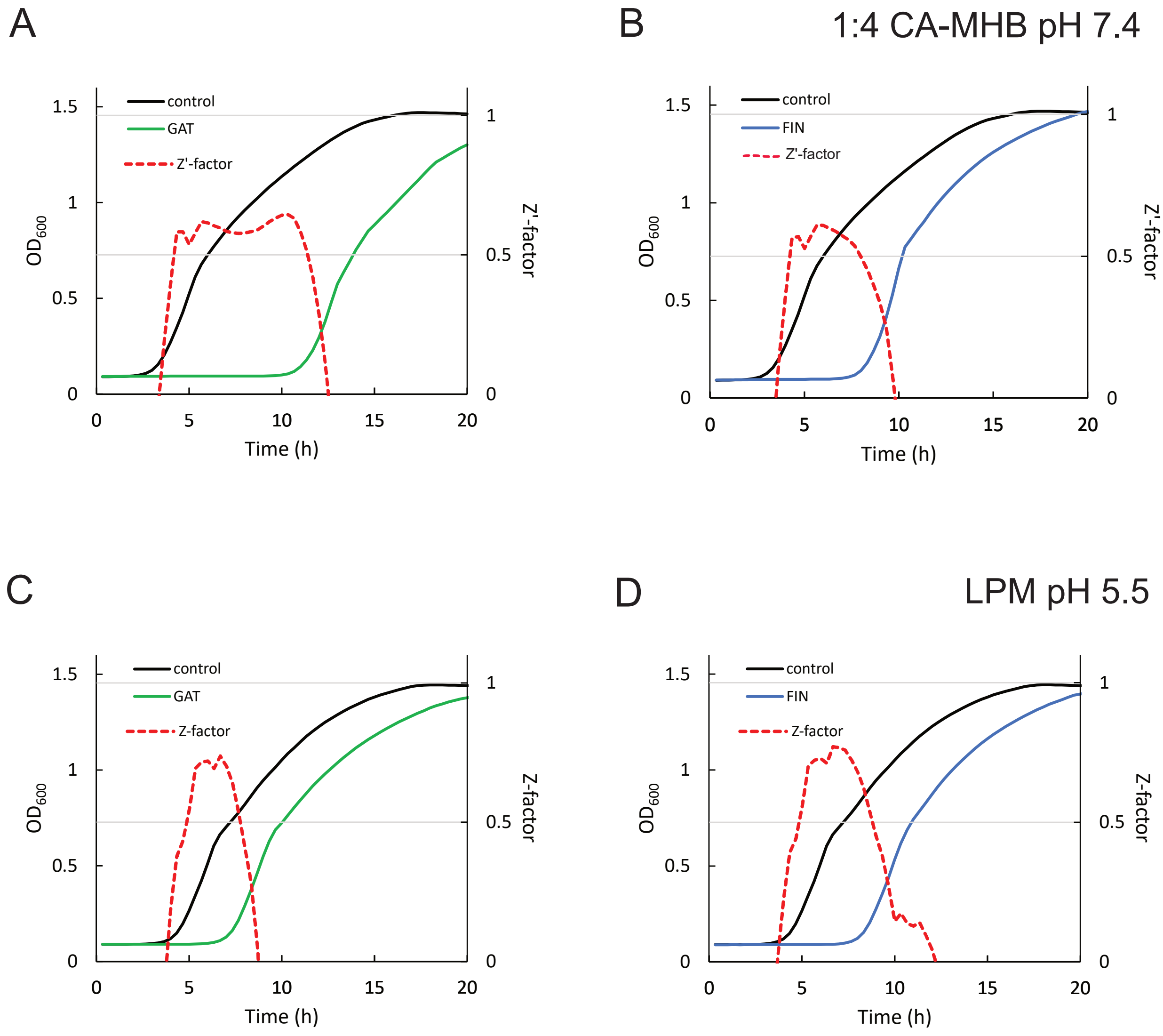

Figure S1. Validation of the screening assay to identify compounds active against non-growing UPEC.

UPEC CFT073 was cultivated in 1:4 diluted CA-MHB (pH 7.4) (A, B) or LPM (pH 5.5) (C, D) for 24 h. Cultures were then treated with 20 µM gatifloxacin (GAT) (A, C), finafloxacin (FIN) (B, D), or incubated without antibiotics as a control in a 96-well plate for 24 h. Regrowth was monitored by measuring OD_{600} after a 2,500-fold dilution into CA-MHB. Each experiment included 12 technical replicates, with mean OD_{600} values indicated by lines. OD600 readings were used to calculate the Z'-factor for the assay.

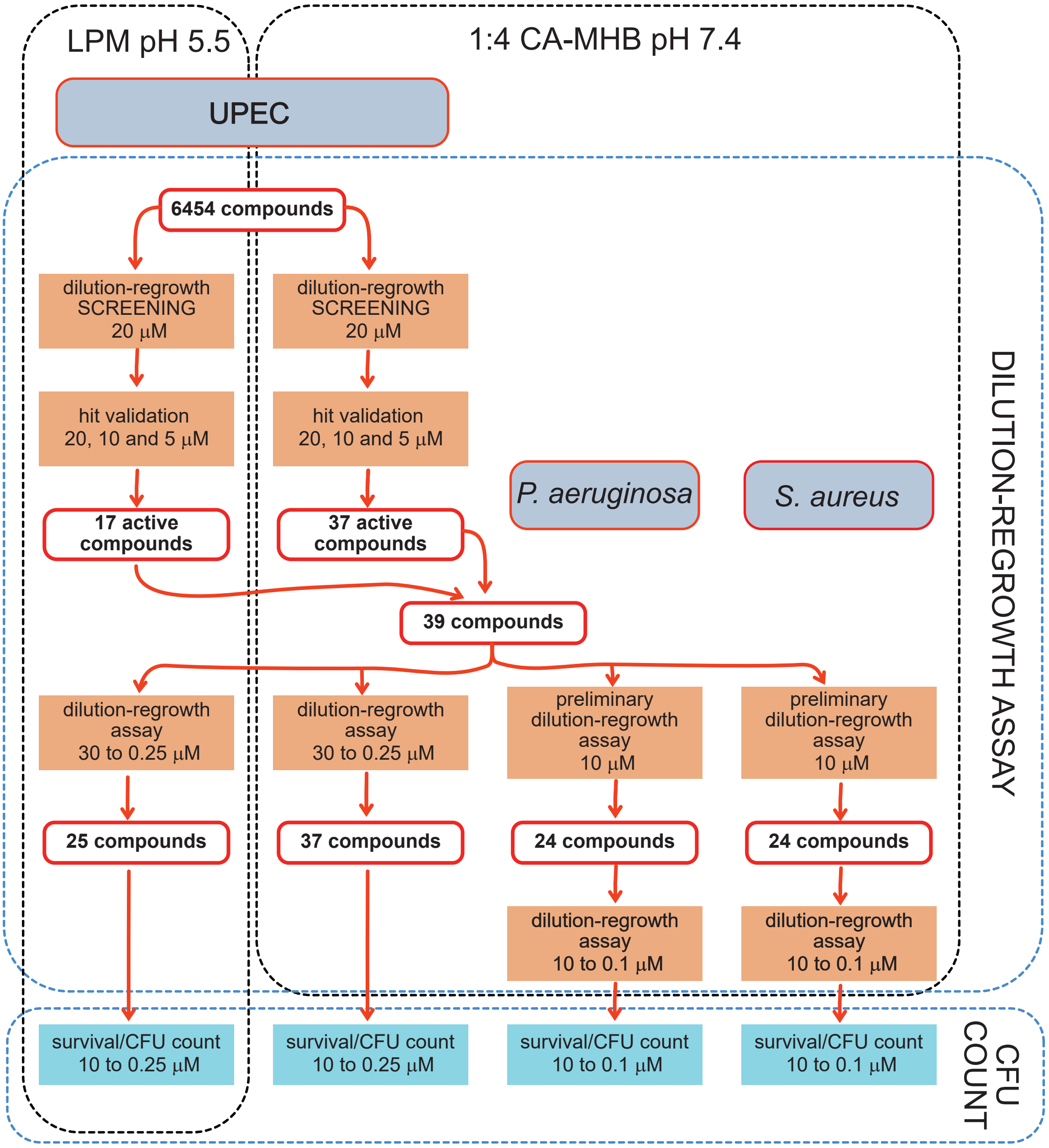

Figure S2. Workflow for the screening and follow-up characterization of compounds active against non-growing bacteria.

The screening of combined Prestwick and SPECS collections identified compounds that delayed the regrowth of stationary phase UPEC CFT073 after treatment in 1:4 diluted CA-MHB (pH 7.4) and LPM (pH 5.5). These hits were validated at three different concentrations. A total of 39 active compounds were tested at concentrations ranging from 30 to 0.25 µM for their ability to postpone UPEC regrowth in both 1:4 CA-MHB (pH 7.4) and LPM (pH 5.5). The verified active compounds were further tested for their bactericidal activity against UPEC in both media. The same set of 39 hit compounds was also tested against non-growing *Pseudomonas aeruginosa* DSM1117 and *Staphylococcus aureus* DSM2569. Based on preliminary dilution-regrowth assay results, the 24 most active compounds against each organism were identified and characterized for concentration-dependent regrowth delay and killing of non-growing *P. aeruginosa* and *S. aureus.*

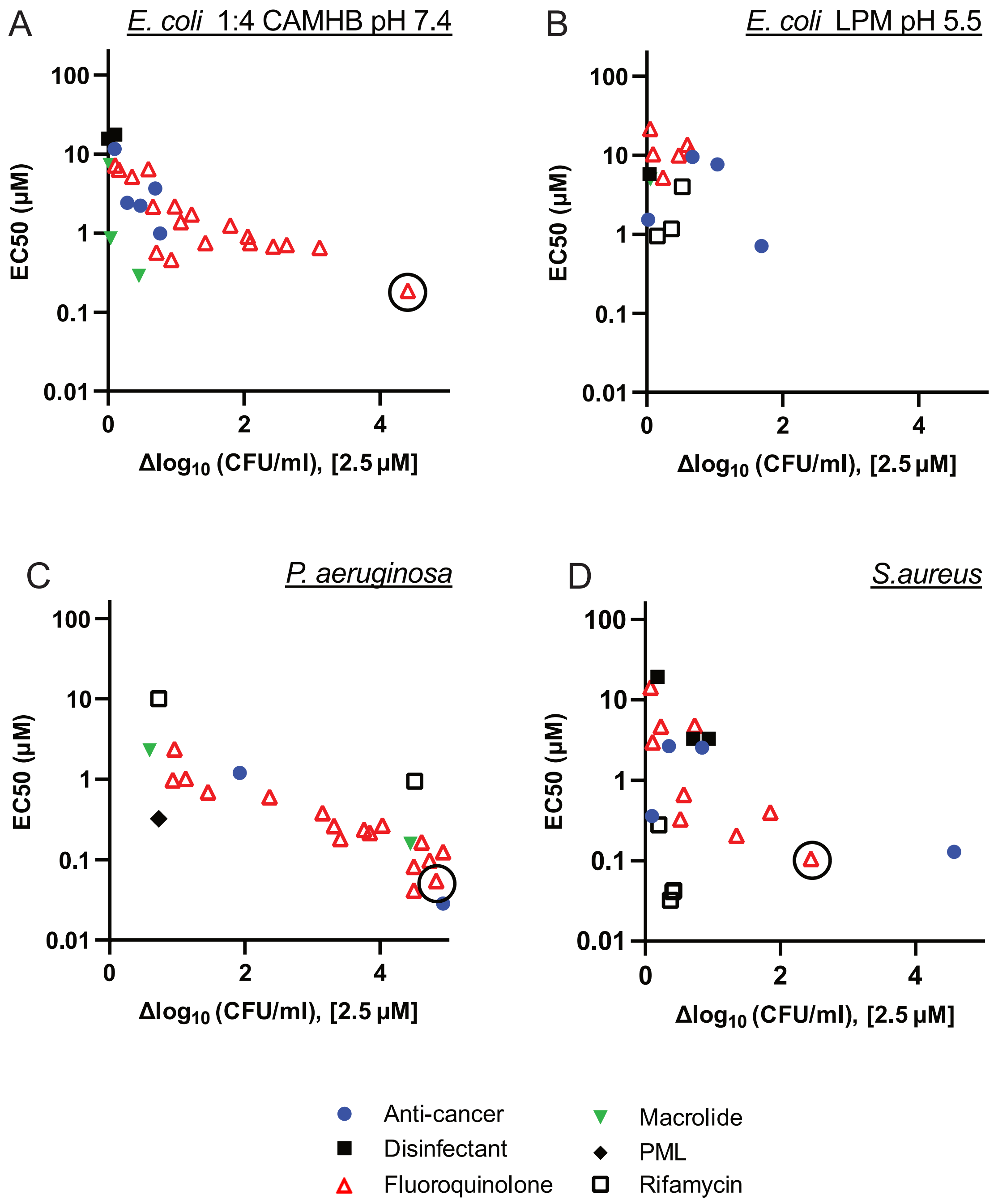

Figure S3. Regrowth inhibition and bactericidal effects of hit compounds on non-growing bacteria.

Bacterial cultures were grown for 24 h, followed by an additional 24 h treatment in 1:4 CA-MHB (pH 7.4) (A, C, D) or LPM (pH 5.5) (B). Regrowth was assessed by measuring OD_{600} after a 2,500-fold dilution into CA-MHB. Regrowth inhibition is represented by EC50 values based on OD600 readings taken 6 h (A, B) or 8 hours (C, D) after dilution. Bacterial killing was evaluated by counting CFUs from samples treated with 2.5 μM of the compounds. The Δlog10 (CFU/ml) indicates the difference between drug-treated samples and the drug-free control. Sitafloxacin is highlighted with a circle.

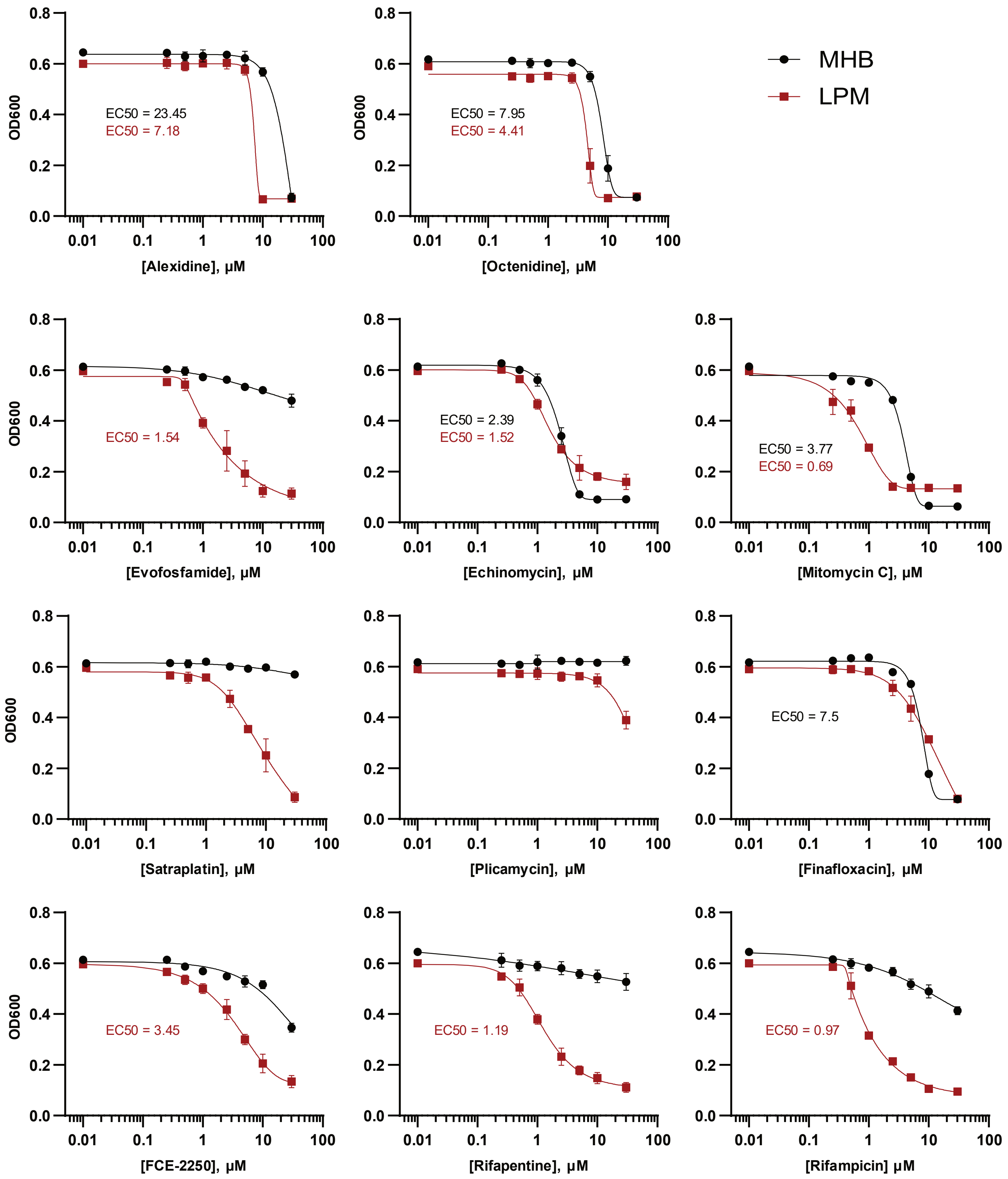

Figure S4. Hit compounds with enhanced activity against non-growing UPEC in acidic medium

Regrowth inhibition curves of the hit compounds with superior activity against non-growing UPEC in LPM (pH 5.5) (red) compared to 1:4 CA-MHB (pH 7.4) (black). Post-treatment regrowth inhibition was assessed by measuring OD_{600} 6 h after a 2,500-fold dilution into CA-MHB. Data are presented as means ±SEM for n=3. A five-parameter logistic equation was used for asymmetric sigmoidal curve fitting and EC_{50} calculation in GraphPad Prism. EC_{50} calculations were omitted when the OD_{600} values did not form a complete sigmoidal curve.

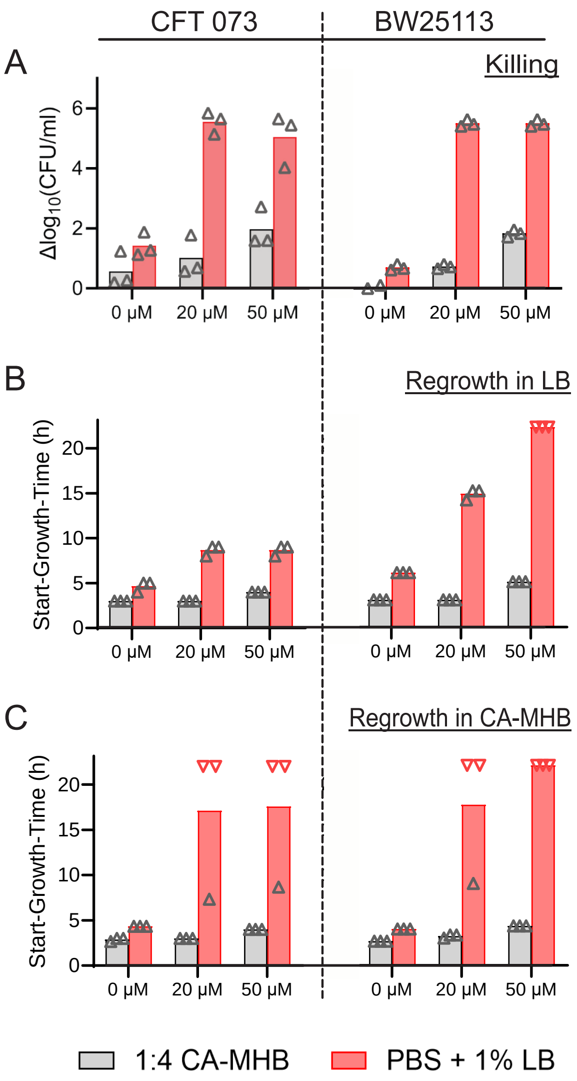

Figure S5. Bactericidal effect of semapimod on non-growing *E. coli* depends on a phosphate buffer-based culture medium.

E. coli strains CFT073 and BW25113 were cultivated in either 1:4 CA-MHB pH 7.4 (gray columns) or PBS supplemented with 1% LB (pink columns) for 24 h, followed by treatment with 20 µM or 50 µM semapimod for an additional 24 h.

A. To estimate survival, samples were serially diluted and spot-plated on LB agar. The differences in CFU/ml before and after treatment are presented. The average log bacterial densities before treatment (log_{10}CFU/ml ± SEM) were 9.97 ± 0.63 for CFT073 grown in 1:4 CA-MHB, 9.49 ± 0.01 for BW25113 grown in 1:4 CA-MHB, 7.74 ± 0.05 for CFT073 grown in PBS + 1% LB, and 7.49 ± 0.07 for BW25113 grown in PBS + 1% LB. Red symbols represent individual experiments where bacterial counts fell below the limit of detection (no colonies from the undiluted sample). The maximum detectable decrease in CFU/ml for these samples is indicated and used for calculating average Δ log_{10}CFU/ml.

B, C. To assess regrowth ability, samples were diluted 2,500-fold into LB broth (B), and into CA-MHB (C). OD_{600} was measured periodically for 22 h post-dilution. The time at which cultures reached an OD_{600} threshold of 0.12 is reported as the Start-Growth-Time (SGT). Red symbols represent individual experiments where cultures did not reach the threshold during 22 h. For these samples, SGT of 22 h is indicated and used for calculating average SGT. Data are presented as means for n=3.

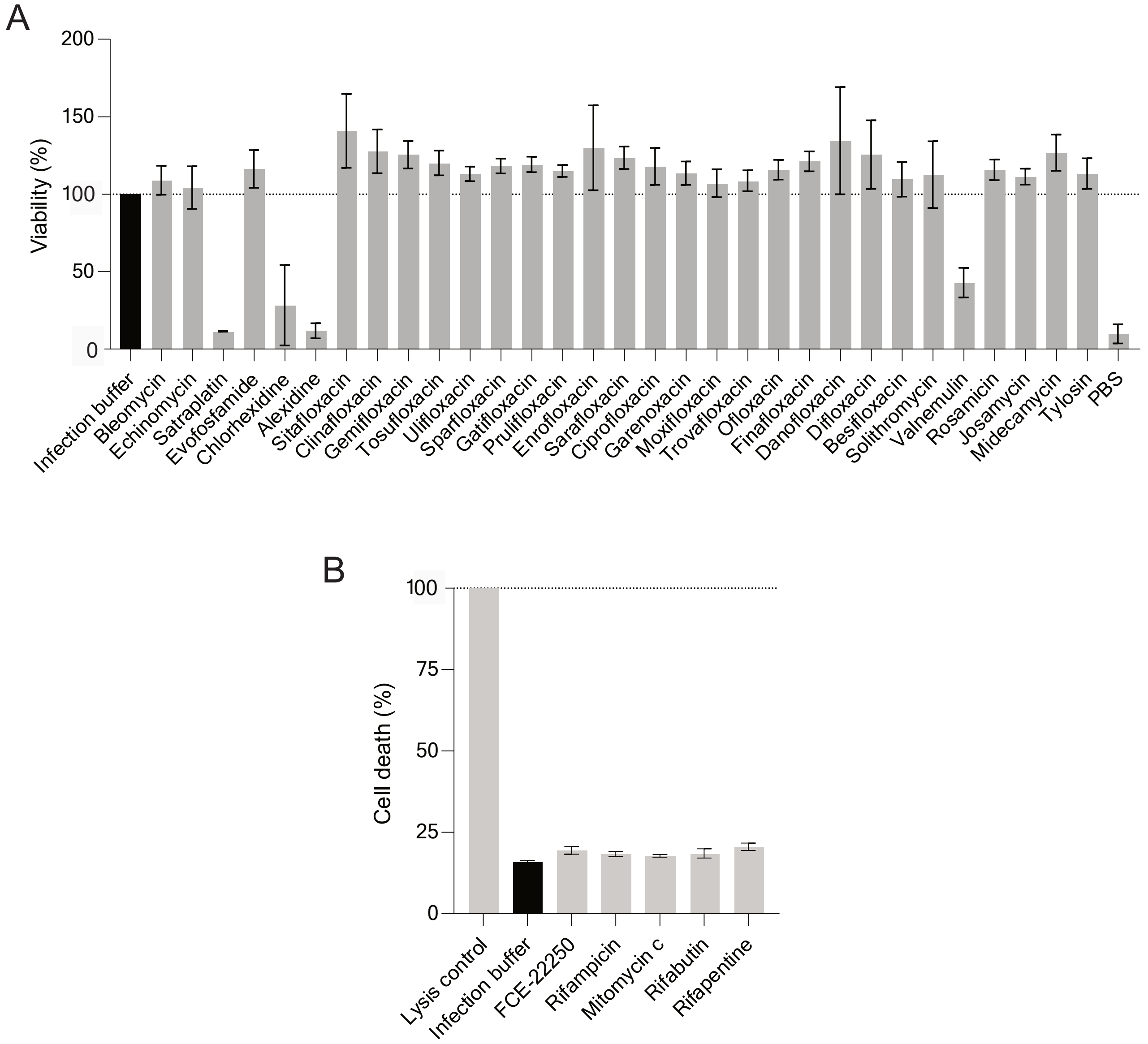

Figure S6. Impact of hit compounds on TC7 cell viability.

A. *Evaluation of toxicity for non-colored compounds using the MTS assay*. TC7 cells were incubated for 12 h with each compound at its minimum inhibitory concentration (MIC) in the absence of bacteria. CellTiter 96 AQueous One Solution Reagent (Promega) was added to cells incubated with infection buffer. Absorbance was measured at 490 nm after 2 h, and values were normalized to the control wells treated with infection buffer only. The experiment was performed twice, with each condition tested in duplicate. Error bars indicate the standard deviation (SD).

B. *Evaluation of toxicity for compounds that absorb light at 490 nm using the LDH release assay.* Cell viability was assessed using the CytoTox-ONE Homogeneous Membrane Integrity Assay kit (Promega) to measure lactate dehydrogenase (LDH) release. Cells in the positive control wells were lysed using lysis solution. Supernatants from each well were incubated with CytoTox 96 Reagent for 10 min, and fluorescence was measured at 560 nm excitation and 590 nm emission. Values were normalized to total lysis controls (representing 100% cell death) and are expressed as means ± SD for n=2.

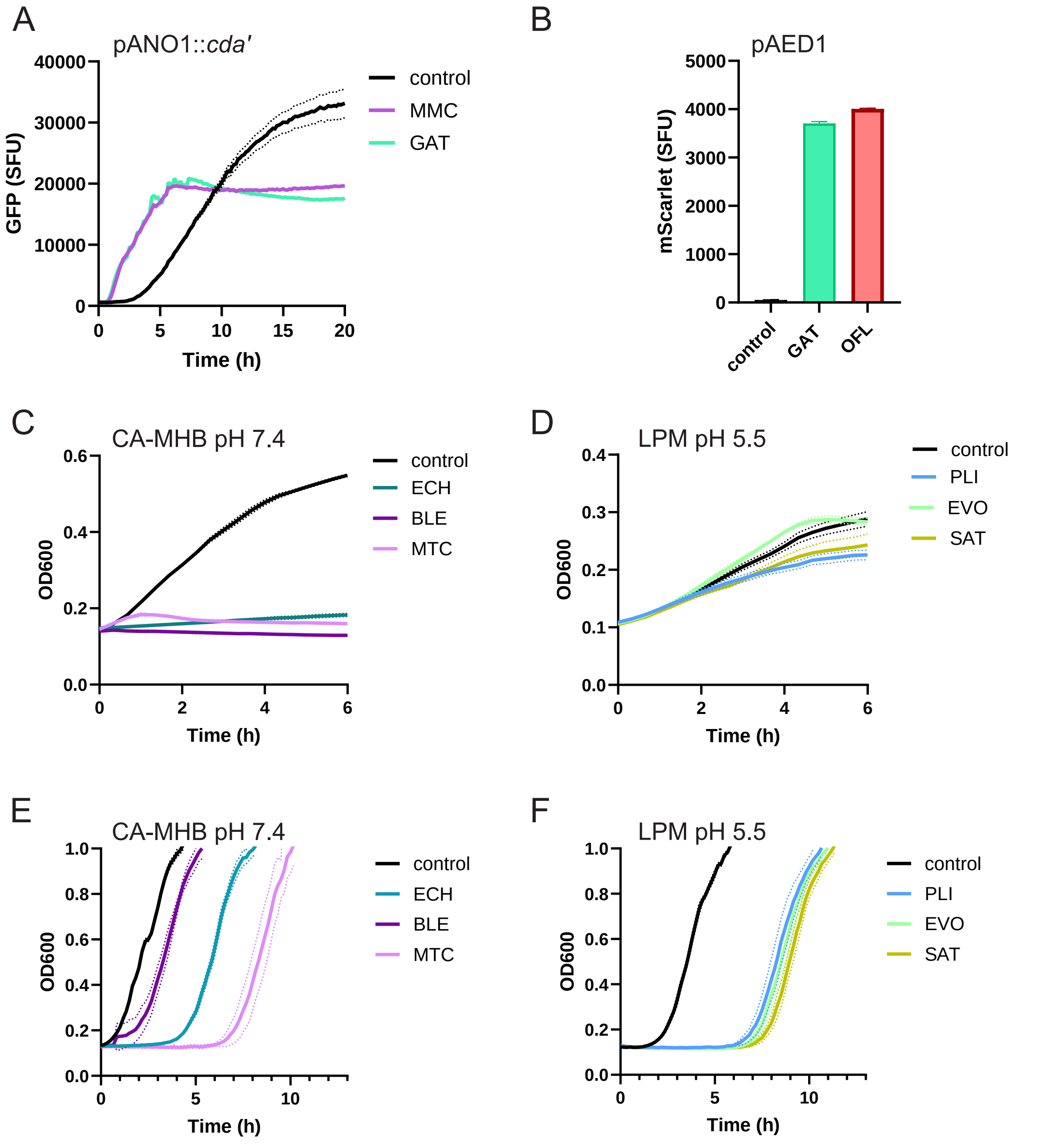

Figure S7. Activity of anti-cancer compounds on UPEC CFT073

A, B. Validation of fluorescent reporters for the SOS response.
A. Cultures bearing the pANO1::*cda'* plasmid were treated with 0.1 µM gatifloxacin (GAT) and 0.5 µM mitomycin C (MMC) or incubated without any drug (control) in a plate reader for 20 h. GFP fluorescence and OD_{600} were recorded every 10 min. Data for the drug-free control represent the means of nine technical replicates ± SEM.
B. Cultures bearing the pAED1 plasmid were treated with 0.3 µM GAT and ofloxacin (OFL) or incubated drug-free (control). mScarlet-I fluorescence and OD_{600} were recorded after 6h of incubation. Data represent the means of four technical replicates ± SEM.
A, B. Specific Fluorescence Units (SFU) were calculated by normalizing arbitrary fluorescence units to cell density (AU/OD_{600}).
C, D. Effect of anti-cancer agents on bacterial growth. OD_{600} was measured for cultures bearing pAED1. Bacteria were grown either in 1:4 CA-MHB (pH 7.4) and treated with 20 µM echinomycin (ECH), bleomycin (BLE), and mitomycin C (MMC) (C), or in LPM (pH 5.5) and treated with plicamycin (PLI), evofosfamide (EVO), and satraplatin (SAT) (D).
E, F. Effect of anti-cancer agents on regrowth following treatment of stationary-phase cultures. Bacteria bearing pAED1 were cultivated in the same media as in panels C and D for 24 h, then treated with the same compounds for an additional 24 h. Bacteria were collected by centrifugation, washed, resuspended, and diluted 1:7 in CA-MHB. OD_{600} of the regrowing cultures was measured.
C-F. Values are presented as means ± SEM for n = 3.

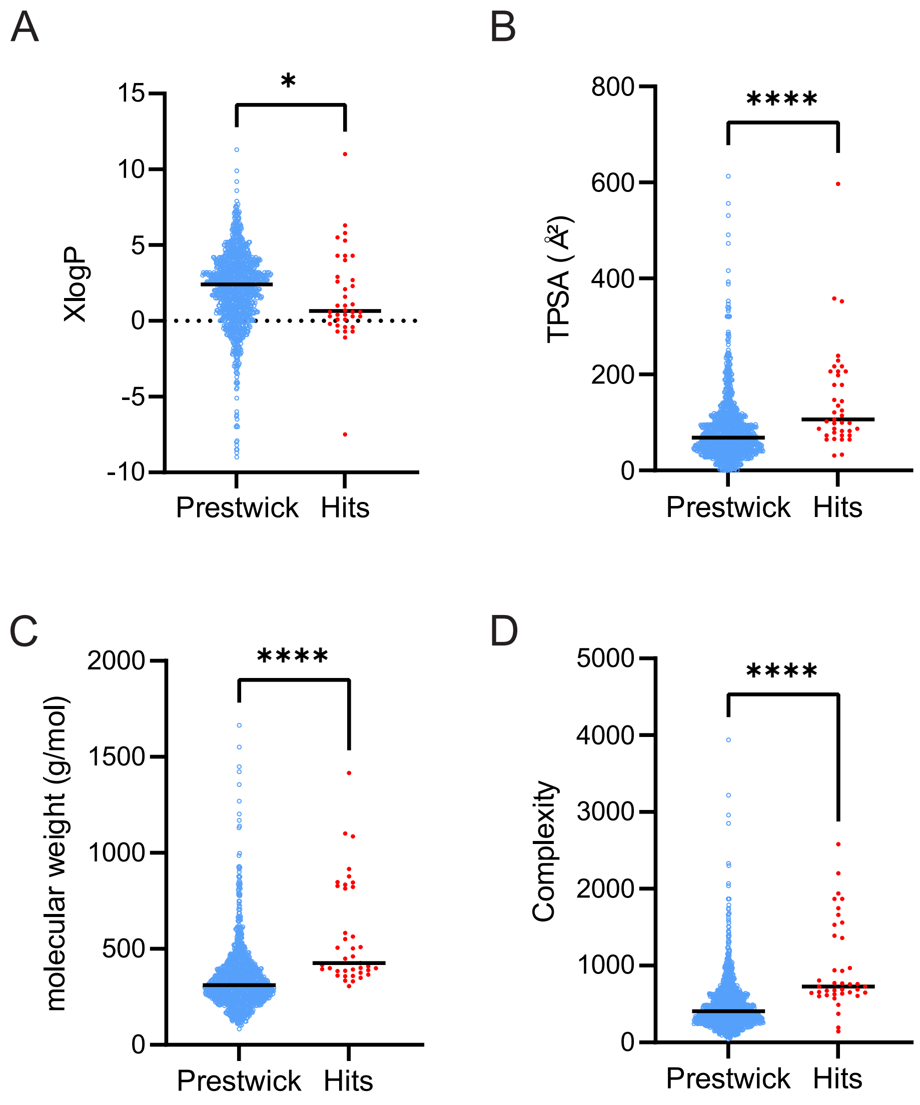

Figure S8. Properties of the hit compounds and the chemicals of the Prestwick collection.

A. Hydrophobicity – partition coefficient, XLogP

B. Topological polar surface area

C. Molecular weight

D. Complexity

Lines indicate median values. The significance of differences between the hit compounds and the Prestwick collection was assessed using a Mann-Whitney test. Four asterisks (****) denote p-values < 0.0001, and one asterisk (*) denotes p-values < 0.05.
